# Blockade of voltage-gated sodium channels inhibits invasion of endocrine-resistant breast cancer cells

**DOI:** 10.3892/ijo.2015.3239

**Published:** 2015-11-09

**Authors:** FATIMA H. MOHAMMED, MAITHAM A. KHAJAH, MING YANG, WILLIAM J. BRACKENBURY, YUNUS A. LUQMANI

**Affiliations:** 1 Faculty of Pharmacy, Kuwait University, Safat 13110, Kuwait; 2 Department of Biology, University of York, Heslington, York, YO10 5DD, UK

**Keywords:** breast cancer, endocrine resistance, voltage-gated Na^+^ channels, Nav1.5, phenytoin, tetrodotoxin, invasion, matrix metalloproteinase

## Abstract

Voltage-gated Na^+^ channels (VGSCs) are membrane proteins which are normally expressed in excitable cells but have also been detected in cancer cells, where they are thought to be involved in malignancy progression. In this study we examined the ion current and expression profile of VGSC (Na_v_1.5) in estrogen receptor (ER)-positive (MCF-7) and silenced (pII) breast cancer cells and its possible influence on their proliferation, motility and invasion. VGSC currents were analysed by whole cell patch clamp recording. Na_v_1.5 expression and localization, in response to EGF stimulation, was examined by western blotting and immunofluorescence respectively. Cell invasion (under-agarose and Matrigel assays), motility (wound healing assay) and proliferation (MTT assay) were assessed in pII cells in response to VGSC blockers, phenytoin (PHT) and tetrodotoxin (TTX), or by siRNA knockdown of Na_v_1.5. The effect of PHT and TTX on modulating EGF-induced phosphorylation of Akt and ERK1/2 was determined by western blotting. Total matrix metalloproteinase (MMP) was determined using a fluorometric-based activity assay. The level of various human proteases was detected by using proteome profiler array kit. VGSC currents were detected in pII cells, but were absent in MCF-7. Na_v_1.5 showed cytoplasmic and perinuclear expression in both MCF-7 and pII cells, with enhanced expression upon EGF stimulation. Treatment of pII cells with PHT, TTX or siRNA significantly reduced invasion towards serum components and EGF, in part through reduction of P-ERK1/2 and proteases such as cathepsin E, kallikrein-10 and MMP-7, as well as total MMP activity. At high concentrations, PHT inhibited motility while TTX reduced cell proliferation. Pharmacological or genetic blockade of Na_v_1.5 may serve as a potential anti-metastatic therapy for breast cancer.

## Introduction

Breast cancer is the most frequently diagnosed neoplasm in women. In the majority of cases, hormonal manipulation by chemical or surgical oophorectomy, or by pharmacological intervention with anti-estrogens such as tamoxifen, remains one of the most effective approaches in the treatment of estrogen receptor (ER)-positive breast malignancies (1,2). Both *de novo* and acquired resistance to endocrine based therapies results from actual or functional loss of ER and is paralleled by cellular transition from an epithelial to a mesenchymal phenotype. Commonly referred to as EMT, this is associated with enhanced proliferative and invasive capacity and results in poor clinical outcome.

Voltage-gated sodium channels (VGSCs) are heteromeric membrane protein complexes containing a single pore-forming α subunit and one or more smaller auxiliary β subunits (3–7). They are classically responsible for initiation and propogation of action potential in excitable cells (8). They effect rapid Na^+^ influx coincident with efflux of intracellular K^+^. In mammals, ten genes encoding VGSCα have been described, nine of which constitute one family with designations of Na_v_1.1 to Na_v_1.9, and Nax (4,6,9–14). These isoforms are encoded by the genes *SCN1A* to *SCN11A*. VGSCα forms an ion pore that differs in its tetrodotoxin (TTX) sensitivity; being sensitive to either nanomolar (Na_v_1.1 to Na_v_1.4, Na_v_1.6 and Na_v_1.7) or micromolar (Na_v_1.5, Na_v_1.8 and Na_v_1.1) concentrations of the toxin (4,9,12,15). VGSCβ are also members of the immunoglobulin superfamily of cell adhesion molecules (CAMs), and responsible for regulating channel gating (16). Four subunits of VGSCβ (β1-β4 encoded by the genes *SCN1B* to *SCN4B*) have been identified in mammals; β1, β1A/B, and β3 are non-covalently bound to the α subunit, while β2 and β4 are linked by disulfide bonds. VGSCβ are multifunctional molecules; they boost channel kinetics, transfer voltage-dependence and expand channel expression in the cell membrane (17). These subunits also promote cell adhesion *in vitro*, both in the presence and absence of the α subunit (7). Thus they are expressed in non-excitable cells such as glia, human endothelial cells and T-lymphocytes (10,11). In addition, they have been found to be overexpressed in various forms of tumors, promoting adhesion, galvanotaxis, motility and invasion, and are therefore associated with poor clinical prognosis (9,11,18–21). For example, in prostate cancer cells, enhanced expression of VGSCα subunit was observed in the highly metastatic cell line MAT-LyLu/PC-3 compared with the poorly metastatic cell line AT-2/LNCaP (18), and tetrodotoxin treatment resulted in significant inhibition of PC3 cell invasion *in vitro* (20).

In breast cancer, the α subunit gene (*SCN5A*) and nNa_v_1.5 protein were found to be upregulated in the highly metastatic *de novo* resistant MDA-MB-231 cells, in contrast to the weakly metastatic MCF-7 (3,22), and are involved in the enhancement of extracellular matrix (ECM) degradation (23), in part through activation of acidic cysteine cathepsins B and S (24).

We have established several endocrine-resistant breast cancer cell lines by shRNA-mediated silencing of ER. These have all undergone EMT, and acquired enhanced proliferative and invasive capacity towards various serum components, insulin-like growth factor-1 and epidermal growth factor (EGF) (25–27). Since VGSC expression/activity was shown to be enhanced in highly metastatic cancer cells, we were interested to test if this channel shows enhanced expression level and activity in our acquired form of endocrine-resistant breast cancer cells (pII), and if their invasive behavior is correlated with blockade of VGSC activity. In the present study, we examined the involvment of VGSCs in these cells, with respect to functions related to tumor progression, either by inhibiting channel activity with pharmacological agents (phenytoin and tetrodotoxin) or through siRNA-mediated reduction of Na_v_1.5 channels. We show for the first time a pro-invasive effect of VGSCs in breast cancer cells with acquired endocrine resistance, modulated in part through enhancement of proteases (cathepsin E and kallikrein 10) and MMP (such as MMP-7) activity.

## Materials and methods

### Cell lines

MCF-7 breast cancer cells were obtained from the American Type Culture Collection (VA, USA). pII cell line (ER silenced) was established in our laboratory by transfection of MCF-7 with ER directed shRNA plasmid as described previously (25,27). For routine culture, all cell lines were maintained as monolayers in advanced Dulbecco's minimum essential medium (DMEM) containing phenol red and supplemented with 5% fetal bovine serum (FBS), 600 μg/ml L-glutamine, 100 U/ml penicillin, 100 μg/ml streptomycin and 6 ml/500 100X non-essential amino acids (all from Invitrogen, CA, USA), and grown at 37°C in an incubator gassed with an atmosphere of 5% CO_2_ and maintained at 95% humidity.

### Drugs, reagents and antibodies

5,5-Diphenylhydantoin sodium salt (PHT; Sigma, USA) was prepared by dissolution in NaOH and stored at −20°C. Tetrodotoxin (TTX; Tocris, UK) was prepared by dissolution in physiological saline solution at pH 7.4, and stored at −80°C. Stock solutions (10 mM) were diluted with PBS to give final concentrations of 100 nM, 1, 10, 50 and 100 μM. EGF powder (Sigma) was re-suspended in 0.1% BSA at 0.1 mg/ml, and stored in aliquots at −20°C. This stock solution was freshly diluted with sterile PBS to give final concentrations of 10, 50 and 100 ng/ml. Phallotoxin and goat anti-rabbit IgG were obtained from Alexa. Anti-Na_v_1.5 anti-body (ab56240) was obtained from Abcam, UK. P-ERK1/2, P-Akt, actin, and anti-HRP-conjugated secondary antibodies were obtained from Cell Signaling, USA.

### Electrophysiology

Membrane currents were recorded from cells grown on glass coverslips using the whole-cell patch clamp technique, as described previously (3,28). A Multiclamp 700B amplifier was used to make recordings in voltage clamp mode, compensating for series resistance by 40–60%. A Digidata 1440A interface (Molecular Devices) was used to digitize currents, which were low-pass filtered at 10 kHz, sampled at 50 kHz and then analyzed using pClamp 10.4 software. Linear leak currents were subtracted using a P/6 protocol (29).

### MTT assay

Approximately 10^4^ cells were seeded into triplicate wells of 12-well plates and allowed to attach overnight. Either vehicle only (control) or VGSC inhibitors PHT or TTX (100 nM-100 μM) were then added to the cells. Growth was assessed after 4 days of incubation. Briefly, 1 ml of MTT [3-(4,5-dimethylthiazolyl-2)-2,5-diphenyltetrazolium bromide] reagent (Promega, USA) (0.5 mg/ml) was added to each well and plates incubated at 37°C for 30 min followed by the addition of 1 ml acidic isopropanol and vigorous re-suspension of the converted blue dye. Absorbance of the suspension was measured at 595 nm with background subtraction at 650 nm.

### Cell motility wound healing assay

pII cells were cultured in 12-well plates to 80–90% confluency. A scratch was created in the cell monolayer using a sterile p100 yellow pipette tip and an image of the scratched area was captured immediately (0 h). The media was then replaced with vehicle (control), phenytoin, or TTX at concentrations of 100 nM, 1, 10, 50 and 100 μM diluted in DMEM. Cells were cultured at 37°C/5% CO_2_. After 24 h, an image was captured of the same scratched area. The width of the scratch at 24 h was calculated as a percentage of the width at 0 h; a minimum of 3 areas along the scratch were measured.

### Agarose invasion assay

Ultra-pure agarose (Invitrogen) was melted in PBS, supplemented with DMEM containing 5% FBS, and allowed to solidify in individual wells of 6-well dishes at room temperature. Once set, cells (4×10^4^) that had been exposed to various concentrations of PHT or TTX, or vehicle (control), were loaded into wells in the agarose formed as previously described (26). Plates were incubated at 37°C in 5% CO_2_ humidified atmosphere. After 24 h, cells that had penetrated into the agarose were manually counted by visual microscopic examination. Random cell invasion was determined as the total number of cells which moved in both lateral directions out of the well. In another experimental setup, cells were treated with EGF (10 ng/ml) in the presence or absence of various concentrations of PHT or TTX and invasion determined. In this latter case, the agarose was mixed with insulin transferrin selenium (ITS) instead of 5% FBS since serum itself has invasive stimulatory components.

### Cultrex BME cell invasion assay

pII cell invasion was also assessed by the Cultrex^®^ 24-well BME cell invasion assay obtained from Trevigen (USA) according to the manufacturer's instructions. In brief, the invasion chamber was coated with 100 μl of 1X basement membrane extract (BME) solution and incubated overnight at 37°C. pII cells serum-starved over-night at 37°C/5% CO_2_, were re-suspended at 10^6^ cells/ml in DMEM (control) or DMEM containing various doses of PHT (1, 10, 50 and 100 μM), and 100 μl of suspension was loaded into the upper chamber. The lower chamber was loaded with 500 μl DMEM supplemented with 10% FBS, as a chemoattractant. Cells were incubated at 37°C, 5% CO_2_ and allowed to migrate from the top chamber to the bottom. After 48 h, liquid from both top and bottom chambers was removed by aspiration and chambers gently washed with 1X cell wash buffer, provided by the supplier. Calcein-AM/cell dissociation solution complex was added to the bottom chamber and left for 1 h at 37°C/5% CO_2_. Cells internalize Calcein-AM and intracellular esterases cleave acetomethylester (AM) moiety generating fluorescent free calcein. Invading cells were determined by recording the fluorescence emission using a microplate reader with a filter set of excitation/emission = 485/535 nm (Cultrex, 2008).

### Confocal microscopy

MCF-7 and pII cells grown overnight at 37°C, 5% CO_2_ in 8-well glass chambered slides (Lab-Tek, USA) were either left untreated or exposed for 30 min to EGF (50 ng/ml) then fixed with 3.7% paraformaldehyde and stained with phallotoxin (green fluorescence) to visualize F-actin, Na_v_1.5 antibody (red fluorescence), and DAPI (blue fluorescence) to visualize the nuclei, and examined by confocal microscopy using a Carl Zeiss LSM 700 microscope.

### Western blotting

pII cells were cultured in 6-well plates to 80% confluence and then serum-starved overnight before addition of either vehicle or EGF (50 ng/ml). After 30-min exposure cells were harvested by scraping into 300 μl of lysis buffer containing 50 mM HEPES, 50 mM NaCl, 5 mM EDTA 1% Triton X-100, 100 μg/ml PMSF, 10 μg/ml aprotinin, and 10 μg/ml leupeptin. Protein was determined by the standard Bradford assay and 6 μg were mixed with an equal volume of 2X SDS and heated at 90°C for 10 min. Lysates were loaded onto a 10% SDS-polyacrylamide gel and electrophoresed at 150 V for 1 h. Proteins were transferred to a nitrocellulose membrane and blocked with 2% BSA for 1 h before being incubated overnight at 4°C with either total or pAkt antibody (1/600 dilution), pERK1/2 antibody (1/1,000 dilution), Na_v_1.5 antibody (1/100 dilution), or actin antibody (1/1,000 dilution) prepared in 2% BSA. The membrane was washed and incubated with anti-HRP-conjugated secondary antibody (1/500 dilution) for 1 h, developed with Super Signal ECL and visualized with Kodak X-ray film.

### Matrix metalloproteinase activity

The general activity of MMPs was determined using a kit from Abcam (cat no. ab112146) according to the manufacturer's protocol. pII cells were seeded into 6-well plates and allowed to grow to 80% confluence. Cells were serum starved overnight, and then either left untreated or exposed to 100 μM phenytoin for 1 h followed by EGF stimulation (100 ng/ml) for 30 min. Then, 25 μl of the media was removed and added to 25 μl of 2 mM APMA working solution and incubated for 15 min at 25°C, followed by addition of 50 μl of green substrate solution. MMP activity was measured at 10-min intervals for 1 h, at 37°C by recording fluorescence emission using a microplate reader with a filter set of excitation/emission = 485/535 nm.

### Proteome profiler analysis

The relative change in 35 human proteases was detected using Proteome Profiler™ human protease array kit (cat no. ARY021B, R&D Systems, Inc., Minneapolis, MN, USA) following the manufacturer's protocol. Briefly, pII cells were cultured in 6-well plates until reaching 80–90% confluency, then serum-starved overnight, and either left untreated (UT, control) or exposed to 50 μM phenytoin for 1 h followed by EGF stimulation (100 ng/ml) for 30 min. Cell lysate was harvested by scraping into 300 μl of lysis buffer containing 50 mM HEPES, 50 mM NaCl, 5 mM EDTA 1% Triton X-100, 100 μg/ml PMSF, 10 μg/ml aprotinin, and 10 μg/ml leupeptin. Protein was determined by the standard Bradford assay. Nitrocellulose membranes with duplicate spots of selected capture antibodies were incubated in 2 ml of array buffer 6 (works as blocking buffer) in 4-well multi-dishes on a rocking platform for 1 h. For each membrane, protein samples (200 μg) were incubated at room temperature with 15 μl of protease detection cocktail for 1 h, before adding onto the membrane, and incubated overnight at 4°C. The membranes were then washed with 1X wash buffer and incubated with streptavidin HRP for 30 min. Following another wash, the membranes were incubated with chemi-reagent mix and exposed for 10–20 min. Spot intensity was quantified using a densitometer and the average of duplicate spots on the membrane was normalized with the average negative control spots according to the manufacturer's protocol.

### siRNA transfection

pII cells were plated in 12-well plates in complete DMEM and incubated for 18 h at 37°C, 5% CO_2_. Transfection was performed using 25 pmol of Na^+^ CP type Vα siRNA (h) obtained from Santa Cruz Biotechnology (cat no. sc-42640). Solution A was prepared following the manufacturer's protocol by diluting 1 μl Stemfect RNA transfection reagent (Stemgent, cat no. 00-0069) into 24 μl buffer. Solution B was made by diluting 2.5 μl of siRNA transfection reagent into 22.5 μl of buffer. Solutions A and B were then mixed, incubated for 15 min, and added dropwise to the cells. After 48–72 h cells were harvested and RNA extracted for determination of *SCN5A* expression by SYBR-Green real-time quantitative PCR as described below.

### RNA extraction

RNA was extracted from transfected pII cells and purified using the RNeasy kit (Qiagen, USA) following the manufacturer's protocol. The concentration and yield of RNA was determined spectroscopically using the Nano-Drop (Pharmacia) and integrity checked by agarose gel electrophoresis.

### Quantitative real-time PCR

RNA was converted to cDNA using a High-Capacity cDNA Reverse Transcription kit from Applied Biosystems. Quantitative PCR was performed in the ABI 7500 FAST thermocycler in a reaction volume of 20 μl using the SYBER green master mix from Invitrogen). Primers for *SCN5A* gene (forward primer 5′-CACGCGTTCACTTTCCTTC-3′, reverse primer 5′-CATCAGCCAGCTTCTTCACA-3′; 208-bp product) and β-actin were synthesized in the HSC Research Core Facility, Kuwait University.

### Statistical analysis

Means of various groups were compared using the Sudent's t-test. Differences were considered significant at p≤0.05.

## Results

### VGSC current in pII and MCF-7 cells

**Figure 1:**
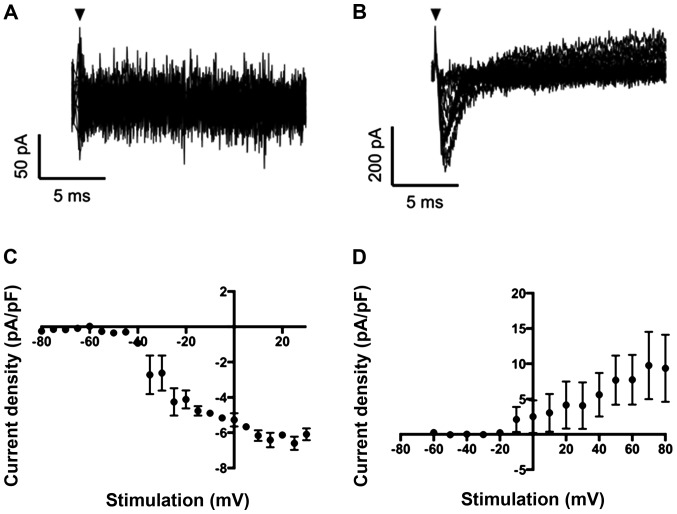
VGSC currents in MCF-7 and pII cells. (A) Representative trace showing that there was no obvious inward current in MCF-7 cells. (B) pII cell exhibiting fast inward VGSC current. (C) Inward and (D) outward current-voltage relationship of pII cells. Cells were held at −120 mV for 250 ms before depolarization to voltages ranging from −80 to +30 mV in 5 mV increments for 50 ms.

Whole-cell patch clamp recording revealed that VGSC currents were absent in MCF-7 cells (n=8 recordings), consistent with previous reports (Fig. 1A) (22). Interestingly, however, ER silencing in pII cells resulted in the upregulation of a fast inward Na^+^ current in 2 of 5 cells recorded (Fig. 1B–D).

### Effect of phenytoin and TTX on pII cell proliferation and motility

**Figure 2:**
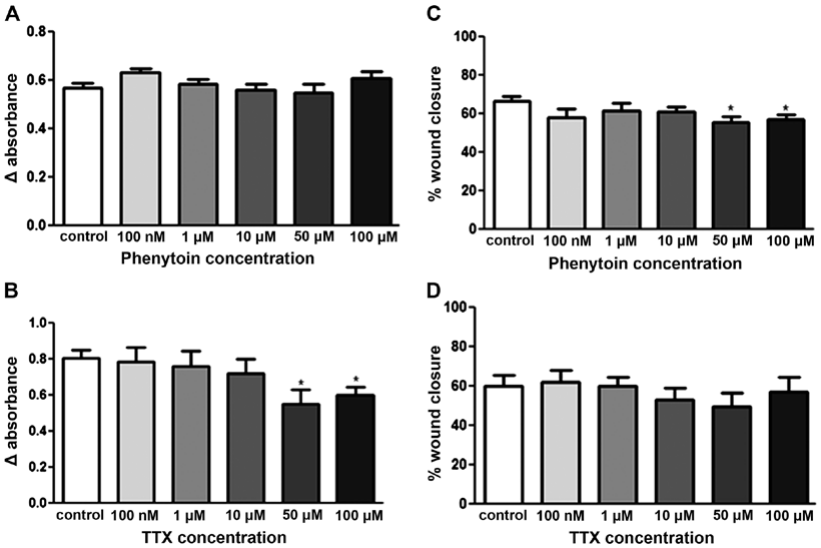
Effect of PHT and TTX on proliferation and motility of pII cells. Cells grown in microtitre plates were either left untreated (control, open bars) or exposed to PHT/TTX at the concentrations indicated. Growth was assessed by MTT assay after 4 days of incubation (A and B). Motility was determined by the wound healing assay after 24 h of incubation (C and D). Histobars represent the mean ± SEM of 4–14 independent determinations, ^*^Significant difference from control with p≤0.05.

The effect of various doses (100 nM-100 μM) of PHT and TTX on pII cell proliferation was assessed using the MTT assay. As shown in Fig. 2A, phenytoin had no effect, while TTX exhibited a small inhibitory effect (25–30%) when used at the higher concentrations (50–100 μM; Fig. 2B). On the other hand, TTX had no effect on cell motility whereas phenytoin had a small (8–10%) inhibitory effect at 50–100 μM (Fig. 2C and D).

### Effect of PHT and TTX on random invasion of pII cells towards serum components

**Figure 3:**
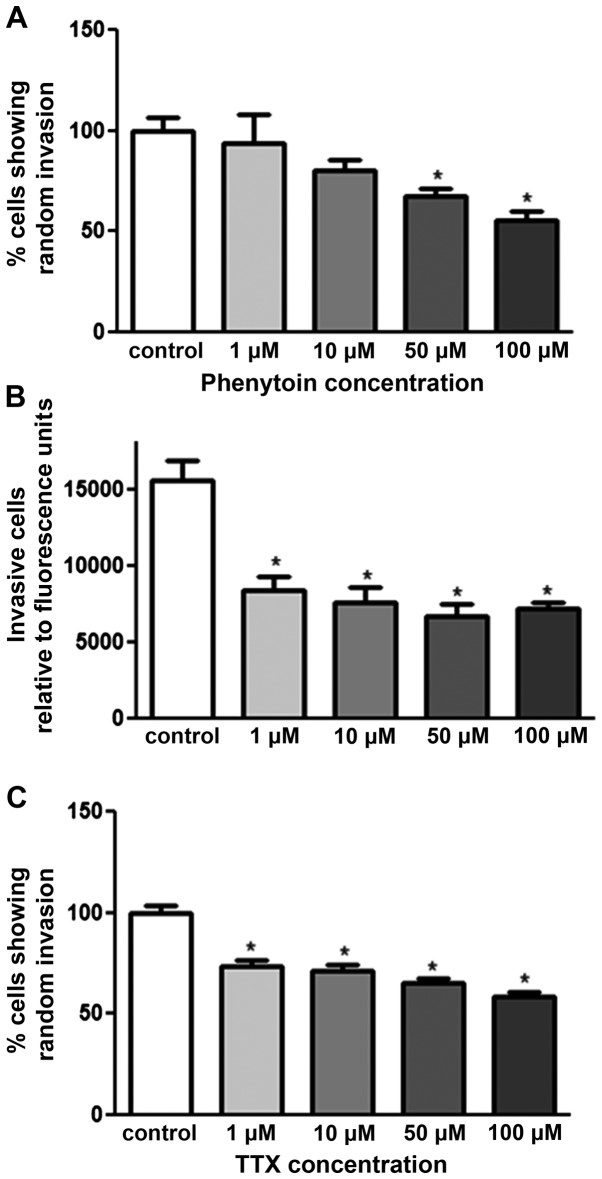
Effect of PHT and TTX on invasion of pII cells towards serum components. Cells were either left untreated (control, open bars) or exposed to various doses of PHT/TTX. The total number of pII cells penetrating into the agarose layer were manually counted as described in Materials and methods [control set as 100%; (A and C)]. (B) The effect of phenytoin on penetration of cells through a basement membrane extract (BME) using the Matrigel assay towards serum components. The y-axis shows arbitrary fluorescence units indicating uptake of Calcein into penetrating cells as described in Materials and methods. Histobars represent the mean ± SEM of 3–18 independent determinations, ^*^Significant difference from control with p≤0.05.

The agarose invasion assay was used to determine the effect of VGSC inhibitors on pII invasion towards serum components. As shown in Fig. 3A and C, both drugs exhibited dose-dependent inhibition; PHT exerted its effect at 50 and 100 μM with 40 and 50% inhibition respectively. TTX exhibited its effect from a lower concentration (1 μM) with 25% inhibition. Matrigel invasion assay was used to confirm the anti-invasive property of PHT. Fig. 3B shows a significant inhibitory effect of PHT at all doses used (1–100 μM) with 50% inhibition.

### Distribution and expression profile of VGSCs in endocrine sensitive and resistant breast cancer cell lines

**Figure 4:**
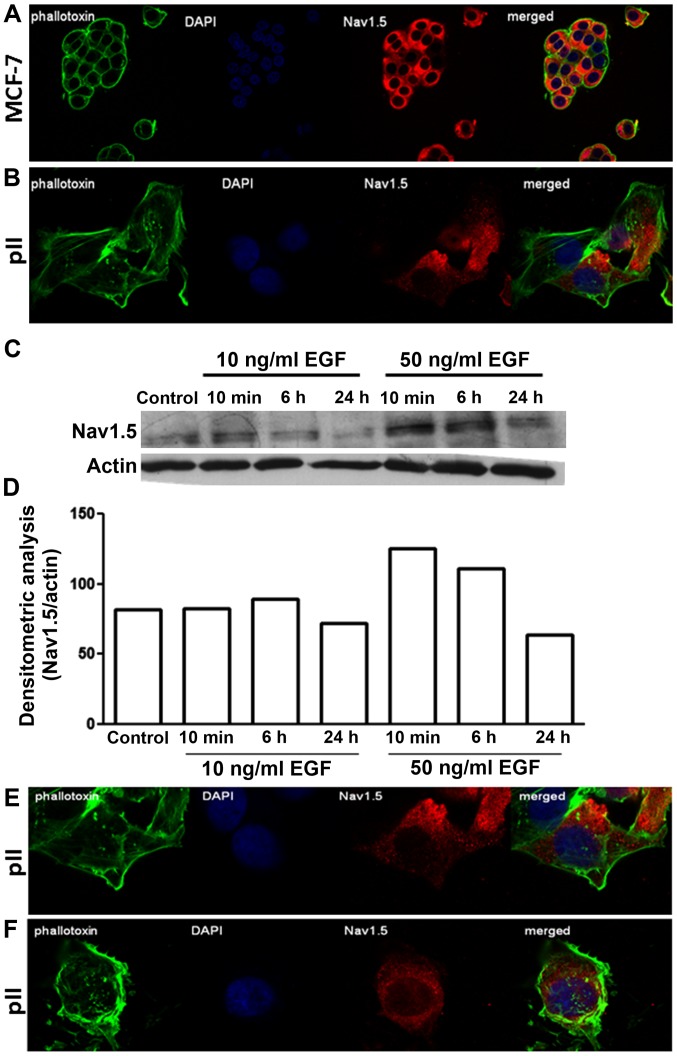
Distribution of VGSCs in MCF-7 and pII cells. Cells were seeded into 8-chambered slides and allowed to grow for 48 h at 37°C/5% CO_2_. Cells were then fixed and stained with Na_v_1.5 antibody (red), phallotoxin (green) and DAPI (blue). (A) MCF-7. (B and E) pII untreated. (F) pII stimulated with EGF 10 ng/ml for 30 min. (C) pII cells serum starved for 2 h and then either left untreated (control) or exposed to 10 or 50 ng/ml EGF and subsequently harvested at 10 min, 6 h and 24 h. Protein lysates were electrophoresed in 10% SDS-polyacrylamide gel, blotted onto nitrocellulose membrane and probed with antisera against Na_v_1.5 and β-actin. (D) Densitometric analysis of the bands in the blot in (C) expressed as a ratio of Nav1.5/actin for normalisation.

Immunofluorescence indicated a similar diffuse cytoplasmic as well as perinuclear distribution of Na_v_1.5 in both MCF-7 and pII cells (Fig. 4A and B). Measurement of total protein by western blotting showed enhanced expression upon EGF stimulation (at 50 ng/ml) in pII cells; the cellular distribution of Na_v_1.5 remained unchanged (Fig. 4C–E).

### Effect of PHT and TTX on invasion of pII cells towards EGF

**Figure 5:**
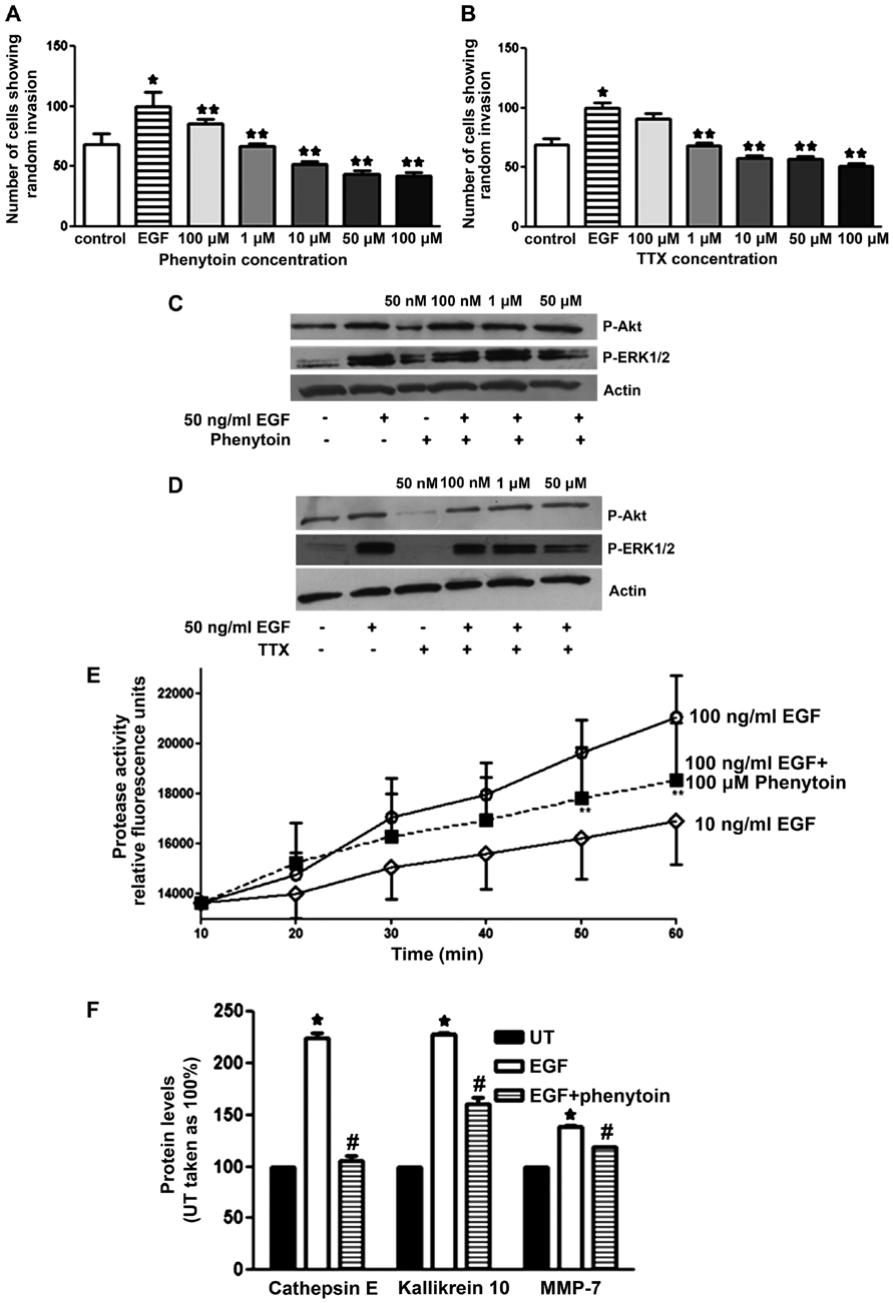
Effect of PHT and TTX on EGF-induced invasion, protease levels, and MMP activity. (A and B) pII cells were either left untreated (control, open bars), treated with EGF (50 ng/ml, hatched bars), or pre-treated with various doses of PHT/TTX plus EGF (solid bars). Total number of cells penetrating into the agarose layer were manually counted as described in Materials and methods. (C and D) pII cells were serum starved overnight and either left untreated or stimulated with EGF in the presence or absence of PHT/TTX. Protein lysates were electrophoresed on SDS-PAGE and proteins transferred onto nitrocellulose membrane and probed with antisera against P-ERK1/2, P-Akt, or actin. (E) pII cells were seeded into 6-well plates and grown until 90% confluent, then serum starved overnight. Cells were then treated with EGF (10 and 100 ng/ml) with or without PHT pre-treatment (50 μM, for 1 h). MMP activity was determined using a fluorogenic substrate (as described in Materials and methods). (F) Protease levels were determined in pII cells; untreated (solid bars), treated with EGF (100 ng/ml, open bars), or treated with phenytoin (50 μM) followed by EGF treatment (100 ng/ml, hatched bars). Histobars represent the mean ± SEM of 3–14 independent determinations. ^*^Significant difference (with p≤0.05) from control; ^#^EGF treatment.

Fig. 5A and B shows a significant increase in the invasive capacity of pII cells towards the well containing EGF compared to vehicle (PBS, hatched bars); this was associated with elevated ERK1/2 phosphorylation and total MMP activity (Fig. 5C and D, second line; and E, open circles). Both drugs (PHT and TTX) showed similar dose-dependent inhibition of the EGF-induced invasion. As shown in Fig. 5B and C (line 6), the anti-invasive property of VGSCs was in part through reducing EGF-induced ERK1/2 (but not Akt) phosphorylation as well as reduction of EGF-induced MMP activity (Fig. 5E). In addition, by using the human protease profiler kit, we observed that EGF stimulation significantly enhanced the levels of cathepsin E, kallikrein-10 and MMP-7 (Fig. 5F) relative to controls. This effect was significantly inhibited by pre-treatment with phenytoin.

### Na_v_1.5 knockdown by siRNA transfection

**Figure 6:**
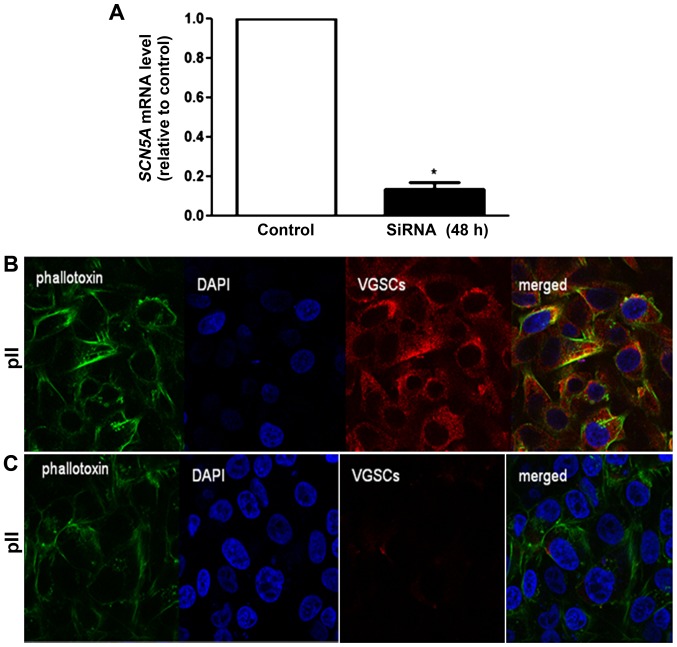
VGSC knockdown by siRNA transfection. (A) pII cells were seeded into 12-well plates, allowed to attach overnight and then either transfected with a scrambled sequence (open bar) or *SCN5A* siRNA (solid bar). RNA was extracted from the cells, converted to cDNA and PCR amplified. Ct values were converted to ratios as described in Materials and methods. Histobars represent mean ± SEM of 3 independent determinations. ^*^Significant difference from control with p=0.026. (B and C) Cells were seeded into a μ-dish^35mm, high^ and incubated at 37°C/5% CO_2_ for 24 h, then transfected with scrambled sequence (B) or *SCN5A* siRNA (C). After 48 h, cells were fixed and stained with Na_v_1.5 antibody (red), phallotoxin (green) or DAPI (blue).

Fig. 6A shows a significant decrease in expression of *SCN5A* mRNA (80%) at 48 h post-transfection with targeting siRNA. Knockdown of Na_v_1.5 protein was confirmed by immunofluorescence with anti-VGSC antisera. Whereas there was clear cytoplasmic as well as perinuclear staining in cells transfected with a scrambled sequence the specific siRNA transfected cells showed almost no signal (Fig. 6B and C). Cytoskeletal staining with phallotoxin was the same in both.

### Effect of SCN5A knockdown on pII cell motility and invasion

**Figure 7:**
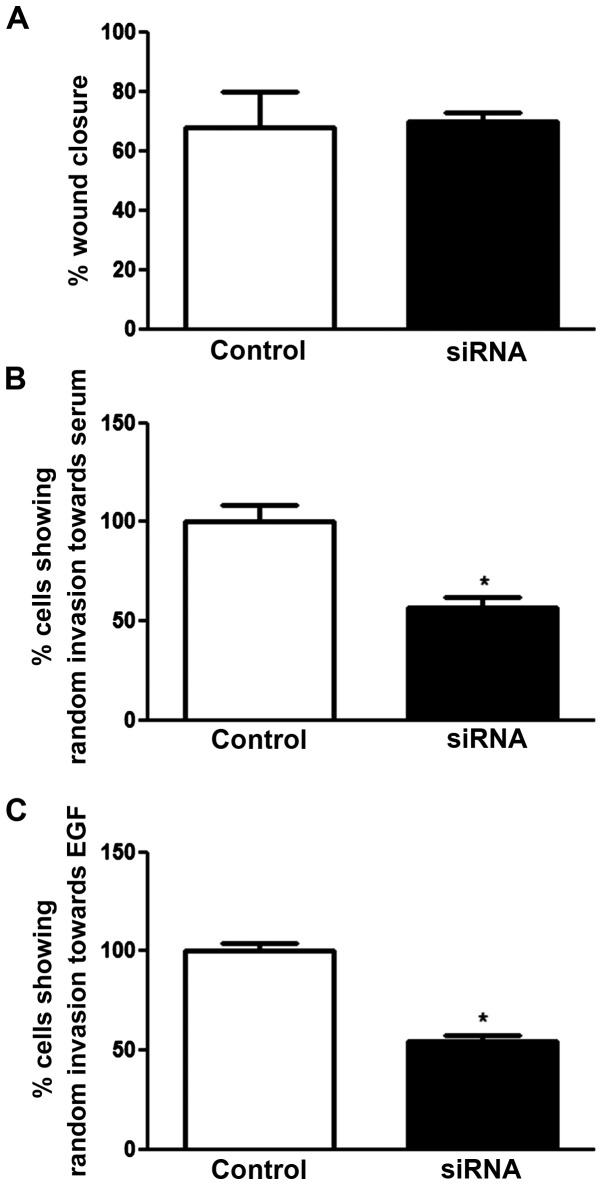
Effect of *SCN5A* siRNA knockdown on pII cell motility and invasion. Cells were either transfected with scrambled sequence (control, open bars) or with *SCN5A* siRNA (solid bars). Motility (A), and invasion towards serum components (B) or EGF (C) was determined as described in Materials and methods. Histobars represent the mean ± SEM of 3–6 independent determinations. ^*^Significant difference from control, with p≤0.05.

As shown in Fig. 7A *SCN5A* mRNA knockdown did not affect pII cell motility compared to control, but significantly inhibited invasion (50%) towards serum components and EGF (Fig. 7B and C).

## Discussion

Although VGSCs are mainly expressed in the plasma membrane of neuronal cells (28), these channel proteins could have roles aside from regulating membrane potential. We showed that Na_v_1.5 is expressed in the cytoplasmic and perinuclear region, as well as in the lamellipodia, of highly invasive breast cancer cells, suggesting a role in cell migration and invasion (3,5–7,29). We have previously documented that the expression of *SCN5A* gene (encoding Na_v_1.5 protein) was significantly increased (by 4-fold) in the highly invasive ER silenced pII cells compared to the ER^+^ MCF-7 cells which have little invasive capacity (27). In view of these findings, we investigated the expression and involvement of VGSCs in pII cells using whole-cell patch clamp recording, pharmacological inhibitors (PHT and TTX) and siRNA-mediated knockdown of Na_v_1.5 protein. Our observations show that VGSC currents were only detectable in pII cells, and not in MCF-7 cells, suggesting a role in modulating various functions such as cell invasion. Upregulation of the *SCN5A* gene in pII cells compared to MCF-7 might explain the absence of the VGSC current in latter cells. In addition, cells that have undergone EMT (due to ER loss) acquire enhanced invasive capacity and upregulate the level/activity of various growth factor receptors, signalling and adhesion molecules, as well as ion channels. VGSCs can promote cell invasion, in part through modulation of EGF-induced ERK1/2 phosphorylation, protease levels, and MMP activity. We found the same pattern of Na_v_1.5 expression in MCF-7 and pII cells; this localization was not changed by EGF stimulation.

Changes in Na^+^ fluxes in metastatic cancer cells have been shown to regulate intracellular pro-invasive signaling cascades, e.g., persistent MAP kinase signaling leading to downstream phosphorylation of ERK and other targets (30,31). Persistent Na^+^ currents may contribute to invasion via several mechanisms, including: a) allosteric regulation of the Na^+^/H^+^ exchanger, NHE1, giving rise to extracellular acidification (23), b) reverse mode of the Na^+^/Ca^2+^ exchanger, NCX, potentiating intracellular Ca^2+^ signaling (32), c) promotion of invadopodia formation via src kinase activity and cortactin phosphorylation (33), and d) regulation of β1-mediated adhesion-dependent migration and invasion (21). These various mechanisms have been reviewed extensively (34,35).

It has been suggested that Fyn kinase activates the fyn-focal adhesion kinase (FAK)-ERK1/2 pathway, leading to neurite outgrowth (36). In MDA-MB-231 breast cancer cells, fyn kinase was shown to co-localize with the β1 subunit of VGSC, and pharmacological, as well as siRNA-mediated fyn inhibition, resulted in inhibition of the β1-mediated process outgrowth (which is proposed to be mediated through ERK1/2 phosphorylation) (21). In Mat-LyLu rat prostate cancer cells, EGF treatment (for 24 h) significantly increased VGSC current density and cell migration. Importantly, EGF treatment in the presence of TTX (a highly selective VGSC blocker) abolished 65% of the potentiating effect of EGF suggesting that a significant portion of the EGF-induced enhancement of migration occurred via VGSC activity (37). In our study, we showed that EGF treatment significantly enhanced VGSC protein expression (Fig. 4) and siRNA-mediated knockdown of VGSC inhibited EGF-induced invasion in pII cells (Fig. 7C), which is consistent with the data obtained from the prostate cancer cells. We also observed a significant inhibition of ERK1/2 phosphorylation by phenytoin and TTX treatment (Fig. 5) consistent with an involvement in VGSC-mediated invasion of pII cells.

Phenytoin is an anti-epileptic and class 1b anti-arrhythmic agent which inhibits the activity of VGSCs (38). Its binding affinity to VGSCs increases when the channels are in the inactivated state (39). Subtypes of VGSCs such as Na_v_1.5 do not reach a complete inactivation state and carry steady-state Na^+^ currents at depolarized potential (40,41). Cancer cells possess a more depolarized membrane potential compared to normal epithelial or excitable cells, suggesting that some permanent Na^+^ current may be involved in invasion and migratory activity (3,42). At concentrations (50 μM) that are used in treatment of epilepsy, phenytoin significantly inhibits both persistent and transient Na^+^ currents in the *de novo* resistant breast cancer cells MDA-MB-231, resulting in reduction of their invasive potential (3,43). Furthermore, phenytoin significantly inhibits growth, invasion and metastasis of orthotopic MDA-MB-231 breast tumors *in vivo* (44). However, phenytoin had no effect on MCF-7 cell migration or invasion (they do not express Na^+^ currents), nor on cell proliferation of either MDA-MB-231 or MCF-7 *in vitro* (3). TTX, also considered a highly specific VGSC blocker, is reported to suppress metastatic behaviour in human breast, prostate and lung cancer cells *in vitro*. Cell proliferation was also not affected by TTX treatment, suggesting involvement specifically in cell invasion (4,8,45). We observed similar effects of PTH/TTX in pII cells as potent anti-invasive agents (Fig. 3) without significantly modulating either cell proliferation or motility. A marginal decrease was seen in cell proliferation at higher doses of TTX (50–100 μM; Fig. 2B), and a marginal decrease in cell motility with higher doses of phenytoin treatment (50–100 μM; Fig. 1C). It should be noted that the anti-invasive property of PHT or TTX was not due to inhibiting cell proliferation since 24–48-h treatment (the time-point used for the invasion assays) did not affect cell proliferation with either drug (data not shown). Differences in effect on motility and proliferation might be due to the lack of specificity of these agents particularly at higher doses. Therefore, the siRNA-approach was used to confirm these findings in a more specific way. siRNA mediated knockdown of VGSCα isoforms has been reported to supress breast cancer cell invasion (4,24,45), which is in aggreement with our data in pII cells (Fig. 6B and C).

In order to metastasize, cancer cells have to degrade the extracellular matrix (ECM) components, and VGSCs have been suggested to play a role in this process through Na^+^/H^+^ exchanger type I (NHE-1) activation. Fraser *et al* (22) showed that VGSCs (specifically Na_v_1.5) increase Na^+^ influx, which in turn activates the NHE-1 present in caveolae. NHE-1 plays a role in Na^+^ influx regulation, leading to extracellular acidification of the tumor microenvironment, resulting in activation of pH-dependent extracellular matrix degradation by cysteine cathepsins B and S, and subsequent enhancement in cell invasion (23). Matrix metalloproteinases (MMPs) are among the proteins involved in invasion by virtue of their ability to degrade various ECM components including collagens, laminin, fibronectin, vitronectin, enactin, tenascin, elastin and proteoglycans (46). They are also thought to play a crucial role in tumor invasion, metastasis, migration and angiogenesis (47,48). Pharmacological blockade of Na_v_1.5 channels in MDA-MB-231 cells result in a significant decrease in MMP-9 mRNA expression and cell invasion (49), which is also in agreement with our finding of increased MMP activity in the presence of EGF (Fig. 4E). We showed that the most significant inhibitory effect of phenytoin was observed on EGF-induced cathepsin E levels (Fig. 4F). Cathepsin E has been suggested as a possible marker for pancreatic tumors (50,51), and interestingly, a recent study demonstrated a positive correlation between enhanced serum levels of cathepsin E and poor clinical prognosis in breast cancer patients. Mice overexpressing cathepsin E demonstrated enhanced tumor growth and metastasis through induction of the EMT process (52).

From a therapeutic viewpoint, VGSC blockade has been reported to relieve severe cancer pain in patients receiving chemotherapy (53–55). In fact, the newly FDA approved drug, Riluzole, blocks VGSC activity and inhibits metabotropic glutamine receptor, and was reported to prevent side effects related to cancer chemotherapy (56). In addition, Riluzole reduced the metabolic activity of tumors in patients with resectable stage III and IV melanoma (57). Furthermore, the use of VGSC blockers during radical prostatectomy minimized cancer recurrence and metastasis (58,59). Recently, it was suggested that further investigation of the FDA-approved VGSC blockers which are already in the market (for other diseases such as epilepsy and arrhythmia, and for inducing local anaesthesia) should be tested for human metastatic diseases. In conclusion, our data suggest a promising anti-metastatic role for VGSC blockers in acquired forms of endocrine resistance in breast cancer.
